# Sexual Relationship Power and Depression among HIV-Infected Women in Rural Uganda

**DOI:** 10.1371/journal.pone.0049821

**Published:** 2012-12-26

**Authors:** Abigail M. Hatcher, Alexander C. Tsai, Elias Kumbakumba, Shari L. Dworkin, Peter W. Hunt, Jeffrey N. Martin, Gina Clark, David R. Bangsberg, Sheri D. Weiser

**Affiliations:** 1 Division of HIV/AIDS, Department of Medicine, University of California San Francisco, San Francisco, California, United States of America; 2 Wits Reproductive Health & HIV Institute, University of the Witswatersrand, Johannesburg, South Africa; 3 Center for Global Health, Massachusetts General Hospital, Boston, Massachusetts, United States of America; 4 Department of Psychiatry, Massachusetts General Hospital, Boston, Massachusetts, United States of America; 5 Department of Medicine, Harvard Medical School, Boston, Massachusetts, United States of America; 6 Mbarara University of Science & Technology, Mbarara, Uganda; 7 Department of Social & Behavioral Sciences, University of California San Francisco, San Francisco, California, United States of America; 8 Department of Epidemiology, University of California San Francisco, San Francisco, California, United States of America; 9 Department of Psychiatry, University of California San Francisco, San Francisco, California, United States; Indiana University and Moi University, United States of America

## Abstract

**Background:**

Depression is associated with increased HIV transmission risk, increased morbidity, and higher risk of HIV-related death among HIV-infected women. Low sexual relationship power also contributes to HIV risk, but there is limited understanding of how it relates to mental health among HIV-infected women.

**Methods:**

Participants were 270 HIV-infected women from the Uganda AIDS Rural Treatment Outcomes study, a prospective cohort of individuals initiating antiretroviral therapy (ART) in Mbarara, Uganda. Our primary predictor was baseline sexual relationship power as measured by the Sexual Relationship Power Scale (SRPS). The primary outcome was depression severity, measured with the Hopkins Symptom Checklist (HSCL), and a secondary outcome was a functional scale for mental health status (MHS). Adjusted models controlled for socio-demographic factors, CD4 count, alcohol and tobacco use, baseline WHO stage 4 disease, social support, and duration of ART.

**Results:**

The mean HSCL score was 1.34 and 23.7% of participants had HSCL scores consistent with probable depression (HSCL>1.75). Compared to participants with low SRPS scores, individuals with both moderate (coefficient *b* = −0.21; 95%CI, −0.36 to −0.07) and high power (*b* = −0.21; 95%CI, −0.36 to −0.06) reported decreased depressive symptomology. High SRPS scores halved the likelihood of women meeting criteria for probable depression (adjusted odds ratio = 0.44; 95%CI, 0.20 to 0.93). In lagged models, low SRPS predicted subsequent depression severity, but depression did not predict subsequent changes in SPRS. Results were similar for MHS, with lagged models showing SRPS predicts subsequent mental health, but not visa versa. Both Decision-Making Dominance and Relationship Control subscales of SRPS were associated with depression symptom severity.

**Conclusions:**

HIV-infected women with high sexual relationship power had lower depression and higher mental health status than women with low power. Interventions to improve equity in decision-making and control within dyadic partnerships are critical to prevent HIV transmission and to optimize mental health of HIV-infected women.

## Introduction

Depression is prevalent among HIV-infected populations throughout the world [1]–[5]. Among HIV-infected individuals in Uganda, 47% screened positive for probable depression [1], far exceeding population-based estimates of depression in the general population [2]–[5]. Multiple factors may contribute to high levels of depression among HIV-infected individuals, including concerns related to HIV-status disclosure [6], perceived HIV-related stigma [7]–[9], lack of social support [10], and the discomfort and fatigue of illness and physical decline [11]–[13]. Among HIV-infected persons, symptoms of depression are associated with reduced quality of life [14], [15], lower uptake of and adherence to antiretroviral treatment [16], [17], immunologic decline [1], [18]–[20], and AIDS-related mortality [12], [20]–[23].

Depression is also particularly common among HIV-infected women, both in Uganda [10] and elsewhere globally [8], [9], [11], [24]–[26]. Depression is associated with increased transmission risk behaviors among women in the general population, as well as elevated secondary transmission risk among HIV-infected women [2], [27], [28]. Similar to gendered risk of acquiring HIV, risk of depression once living with HIV/AIDS may be partially attributable to gender inequalities in relationship power. This hypothesis is supported by several lines of evidence. First, low sexual relationship power is associated with experiences of intimate partner violence [29] which is itself a robust predictor of depression among women [30]. Second, analyses of data collected from samples of non-HIV-infected persons have linked powerlessness to depression among women experiencing intimate partner violence [27], [31], and among young women [27], [29]. Studies have found other negative health impacts of low sexual power among HIV-infected women, including malnutrition and worse HIV treatment outcomes [32], [33].

While the presence or absence of intimate partner relationships [34], [35] and other social ties [36] are well recognized as key contributors to mental health, less research has explored how power dynamics of intimate partnerships influence mental health [37], [38]. Notably, little work has focused on elucidating how sexual relationship power influences mental health, particularly among HIV-infected women, one of the most vulnerable subgroups in resource-limited settings [39]. We therefore undertook a study to examine whether sexual relationship power is associated with symptoms of depression and mental health status among HIV-infected women in rural Uganda. We also assessed the extent to which the relationship control or decision-making dominance, subscales of the SPRS, were associated with depression.

## Methods

### Participants

The study took place in Uganda within the rural Mbarara District (population 427,000). The town of Mbarara (population 82,000) is located 275 kilometers southwest of Kampala. The main local language is Runyankole, and the majority of residents are ethnically Ankole. The Uganda AIDS Rural Treatment Outcomes (UARTO) prospective cohort study was initiated in July 2005. Participants were recruited from the Mbarara Regional Referral Hospital Immune Suppression Syndrome (ISS) Clinic, which dispenses free HIV antiretroviral therapy (ART) to HIV-infected persons in southwestern Uganda [40]. Participants were included if they were ART-naïve, older than 18 years of age, lived within 20 km of the ISS Clinic, and were initiating free ART. Beginning in August 2007, the study questionnaire was expanded with the addition of measures of sexual relationship power. Data for this paper were drawn from the first two quarterly study visits of all female participants who consented to the UARTO parent study.

### Data collection

Study interviews were conducted using standardized interviewer-administered instruments and blood draws to collect data on sexual relationship power, health behaviors, mental health and other socio-demographic and clinical variables. Surveys and written consent forms were translated into the native language of Runyankole, back translated into English, and pilot-tested to ensure accuracy. All instruments were administered in the field by a native Runyankole speaker. We used standard procedures for data entry and quality control.

### Ethics Statement

Written consent was obtained from all study participants. For participants who could not read or write, they were allowed to mark consent forms with an “X”. Ethical approval for all study procedures was obtained from the University of California San Francisco Committee on Human Research, the Partners Human Research Committee, and the Institutional Review Committee at Mbarara University of Science and Technology.

### Measurements

Our primary predictor, sexual relationship power, was measured at baseline using the sexual relationship power scale (SPRS) [41], a 22-item scale that has been previously used in research conducted in black African populations [42], [43]. The SRPS was developed based on Connell's Theory of Gender and Power [44], which posits that gender imbalances within the household are partly responsible for men's disproportionate power over decision-making. The SRPS contains two subscales: Relationship Control and Decision-Making Dominance. The Relationship Control subscale is comprised of fourteen questions rated on a 4-point Likert-type scale designed to assess the extent to which women can exert sexual and emotional autonomy (e.g., “If I asked my partner to use a condom, he would get angry.”). The Decision-Making Dominance subscale is comprised of eight questions designed to assess the balance of decision making power within the relationship (e.g., “Who usually has more say on whether you have sex?”). Responses are summed and normalized to a range of 1 to 4, with higher scores indicating greater relationship power. As suggested by Pulerwitz et al. [41], scale scores were split into tertiles representing ‘low’, ‘medium’ and ‘high’ power. Both subscales had excellent internal reliability (Relationship Control Cronbach's alpha = 0.95, Decision-Making Dominance alpha = 0.92), as did the SRPS scale as a whole (Cronbach's alpha = 0.96).

Our primary outcome was depression symptom severity, measured as a continuous variable in primary analyses using a modified version of the Hopkins Symptom Checklist for Depression (HSCL-D) [45]–[47]. For this analysis, we used HSCL-D measurements from two time points: at baseline (*t*) and 3 months after baseline (*t*+1). This fifteen-item measure has been widely used and validated in East African settings [10], [48], and achieved a high degree of internal consistency in our sample (Cronbach's alpha = 0.86). Our secondary outcome was a measure of mental well being among HIV-infected persons, namely the Mental Health Summary (MHS) of the 35-item Medical Outcomes Study HIV Health Survey (MOS-HIV) [49]–[52]. The MHS is derived from multiple domains of functioning, including mental health, health distress, quality of life, cognitive function, vitality, and social function. Validation studies have been conducted in numerous settings [49]–[52], including East Africa [53].

### Statistical Analysis

We used multivariable linear regression to measure the associations between sexual relationship power and both (a) depression symptom severity (HSCL) and (b) mental health status (MHS). Depression was specified as a continuous variable, and estimates were adjusted for potential confounders, including age, CD4+ T-lymphocyte cell count, educational level, marital status, positive screen for hazardous drinking as measured by the AUDIT-C [54], [55], tobacco use, history of a WHO stage 4 clinical condition, and length of time on ART. For ease of exposition we used logistic regression to estimate the association between probable depression (using the conventional threshold of 1.75 [56]) and sexual relationship power, while adjusting for the same covariates as specified above. To determine whether different aspects of relationship power were differentially associated with the outcomes of interest, we repeated the analyses using the relationship control and decision-making dominance sub-scales as covariates.

To assess the extent to which our estimates could be driven by reverse causality, we lagged covariates by three months. For this, we used data from two time points. In the first lagged model, we estimated the association between SRPS at time *t* and depression at time *t+1*. In the second lagged model, we estimated the association between depression at time *t* and SRPS at time *t+1*. We repeated these analyses to assess directionality between SRPS and MHS.

## Results

Among the 325 eligible women, only the 270 participants (83.0%) who had complete information on all variables were included in this analysis (Table 1). There was no more than 6% missing data on any specific variable, with percent missingness less than 2% for most variables. There were no statistically significant differences between the 45 excluded individuals and the 270 included individuals in terms of any of our key predictors and outcomes of interest. The median age was 34 years (interquartile range [IQR] = 28–38 years). Approximately one-third of women were married at baseline, less than one-quarter had secondary education, and 34.8% of women were unemployed. Women lived a median of 45 minutes travel from the clinic, with some participants reporting travel times of up to 7 hours. Sixty-four (23.7%) women reported symptoms consistent with probable depression. The median MHS score was 46.7 (IQR, 38.5 to 53.3).

**Table 1:** Descriptive Statistics.

	All participants (N = 270)
*Characteristics*	Median (IQR)
***Sexual Relationship Power Scale (SRPS)***	2.46 (2.10 to 2.85)
***Sociodemographic Characteristics***	
Age	34 (28 to 38)
Married	104 (38.5%)
Household size	3 (1 to 5)
Secondary education	62 (23.0%)
Unemployed	94 (34.8%)
Asset Index	−0.17 (−1.40 to 1.44)
Distance to Clinic	0.75 (0.5 to 1)
***Clinical Characteristics***	
HSCL	1.34 (1.12 to 1.75)
Probable depression (HSCL>1.75)	64 (23.7%)
Mental Health Status	46.7 (38.5 to 53.3)
ART naive at baseline)	178 (54.8)
Heavy drinking (AUDIT-C)	18 (6.7%)
Tobacco Use (ever)	36 (13.3%)
WHO clinical stage IV at baseline	82 (30.4%)
Previously on ART	121 (44.8%)
CD4 count at baseline	207 (136 to 308)

IQR: Inter quartile range, HSCL: Hopkins Symptomatic Check List, ART: Antiretroviral Treatment; AUDIT-C: Alcohol Use Disorders Identification Test.

Sexual relationship power was associated with depression symptom severity in both unadjusted and adjusted models (Table 2). Specifically, in the models with contemporaneous covariates and outcomes, women with either moderate (*b* = −0.21; 95% confidence interval [CI], −0.36 to −0.07) or high relationship power (*b* = −0.21; 95% CI, −0.36 to −0.06) had lower depression symptom severity compared to women with low relationship power. Other correlates of depression in adjusted analyses included being ART naive at baseline and travelling a greater distance to the clinic. When the outcome was specified as a binary variable indicating probable depression, women with moderate (adjusted odds ratio [AOR] = 0.46; 95% CI, 0.22–0.97) and high (AOR = 0.44; 95% CI, 0.20–0.93) relationship power were less likely to meet screening criteria for probable depression compared to women with low SRPS scores. In the lagged-covariate specification, SPRS at time *t* was associated with depression at time *t+1*.

**Table 2:** Unadjusted and adjusted associations between depression severity and sexual relationship power (N = 273).

	Depression severity (HSCL as continuous scale) Regression estimate *b* (95% confidence interval [CI])		
*Characteristic*	Univariable	Multivariable (Contemporaneous)	Multivariable (Lagged)
***Sexual Relationship Power Scale (SRPS)***			
Low	Ref	Ref	Ref
Moderate	−0.32 (−0.47 to −0.17)***	−0.21 (−0.36 to −0.07)**	−0.12 (−0.22 to −0.01)*
High	−0.29 (−0.44 to −0.14)***	−0.21 (−0.36 to −0.06)**	−0.10 (−0.20 to 0.01)
***Socio-demographic Characteristics***			
Age	−0.01 (−0.05 to 0.03)	0.02 (−0.02 to 0.06)	0.02 (−0.01 to 0.05)
Married	−0.03 (−0.16 to 0.10)	0.04 (−0.08 to 0.17)	0.09 (0.00 to 0.18)
Household size	0.00 (−0.03 to 0.02)	−0.01 (−0.03 to 0.01)	−0.00 (−0.02 to 0.01)
Educated more than secondary school	−0.10 (−0.25 to 0.05)	−0.05 (−0.19 to 0.09)	−0.07 (−0.17 to 0.03)
Unemployed	−0.02 (−0.15 to 0.12	−0.05 (−0.18 to 0.07)	−0.03 (−0.12 to 0.06)
Distance to Clinic	0.08 (0.01 to 0.15)*	0.09 (0.02 to 0.15)*	0.03 (−0.02 to 0.08)
***Clinical Characteristics***			
Heavy drinking (AUDIT-C)	0.30 (0.05 to 0.55)*	0.13 (−0.11 to 0.38)	0.16 (−0.02 to 0.34)
Tobacco Use	0.08 (−0.10 to 0.27)	0.02 (−0.16 to 0.19)	−0.04 (−0.16 to 0.09)
WHO Stage IV clinical condition	0.02 (−0.12 to 0.15)	0.05 (−0.07 to 0.18)	0.03 (−0.06 to 0.12)
CD4 count	−0.03 (−0.08 to 0.01)	0.02 (−0.02 to 0.07)	0.04 (0.01 to 0.07)*
Previously treated with ART	−0.39 (−0.51 to −0.27)***	−0.37 (−0.51 to −0.24)***	−0.19 (−0.29 to −0.09)***
*Constant*	*-*	*1.66*	*1.2*

* P<0.05;

** P<0.01;

*** p<0.001.

HSCL: Hopkins Symptomatic Check List; AUDIT-C: Alcohol Use Disorders Identification Test; WHO: World Health Organization; ART: Antiretroviral Treatment.

Both moderate and high sexual relationship power were also associated with MHS (Table 3). In the models with contemporaneous covariates and outcomes, women with moderate relationship power had better mental health status (*b* = 3.38; 95% CI, 0.08 to 6.68) compared to women with low relationship power. Women with high relationship power also had better mental health status (*b* = 3.03; 95%, −0.32 to 6.39), but the association was not statistically significant. Similar to the analysis of depression above, in the lagged-covariate specification, SRPS at time *t* was associated with MHS at time *t+1*.

**Table 3:** Unadjusted and adjusted associations between mental health and sexual relationship power (N = 273).

	Mental Health Status (MHS as continuous scale) Regression estimate *b* (95% confidence interval [CI])		
*Characteristic*	Univariable	Multivariable (Contemporaneous)	Multivariable (Lagged)
***Sexual Relationship Power Scale (SRPS)***			
Low	Ref	Ref	Ref
Moderate	6.40 (2.91 to 9.90)***	3.38 (0.08 to 6.68)*	3.47 (0.72 to 6.23)*
High	5.55 (2.03 to 9.06)**	3.13 (−0.21 to 6.46)	2.81 (0.13 to 5.49)*
***Socio-demographic Characteristics***			
Age	0.81 (−0.10 to 1.73)	−0.03 (−0.91 to 0.85)	−0.50 (−1.23 to 0.24)
Married	−2.00 (−5.02 to 1.01)	−3.23 (−6.10 to −0.36)*	−1.72 (−4.07 to 0.63)
Household size	0.03 (−0.53 to 0.58)	0.10 (−0.42 to 0.62)	0.25 (−0.19 to 0.67)
Educated more than secondary school	0.78 (−2.72 to 4.28)	0.27 (−3.41 to 2.86)	−0.50 (−3.07 to 2.06)
Unemployed	−1.92 (−5.01 to 1.16)	−0.30 (−3.13 to 2.54)	1.14 (−1.21 to 3.49)
Distance to Clinic	−1.37 (−3.05 to −0.30)	−1.85 (−3.35 to −0.35)*	−0.75 (−1.96 to 0.46)
***Clinical Characteristics***			
Heavy drinking (AUDIT-C)	−48.55 (−14.33 to −2.69)**	−4.15 (−9.57 to 1.26)	−3.82 (−8.25 to 0.61)
Tobacco Use	−0.66 (−4.99 to 3.67)	0.07 (−3.87 to 4.01)	1.60 (−1.67 to 4.86)
WHO Stage IV clinical condition	−0.48 (−3.68 to 2.72)	−1.85 (−4.73 to 1.02)	0.19 (−2.14 to 2.51)
CD4 count	1.69 (0.72 to 2.65)**	0.00 (−0.01 to 0.01)	0.00 (−0.01 to 0.01)
Previously treated with ART	11.25 (8.61 to 13.89)***	10.02 (7.02 to 13.02)***	3.44 (1.00 to 5.89)**
*Constant*	*-*	*41.12*	*48.87*

* P<0.05;

** P<0.01;

*** p<0.001.

MHS: Mental Health Status; AUDIT-C: Alcohol Use Disorders Identification Test; WHO: World Health Organization; ART: Antiretroviral Treatment.

Both subscales of the SRPS were associated with depressive symptoms. Women with both moderate (*b* = −0.31; 95% CI, −0.45 to −0.17) and high Relationship Control (*b* = −0.19; 95% CI, −0.33 to −0.05) had lower depression symptom severity compared to women with low relationship control. Similarly, women with moderate Decision-Making Dominance had lower depression symptom severity (*b* = −0.17; 95% CI, −0.31 to −0.03). High decision-making dominance approached, but did not reach, statistical significance (p = 0.09).

Finally, in order to assess the potential bi-directionality of the relationship between SRPS and the outcomes, we fit regression models with the outcomes specified as the primary exposure, and these were also lagged. Depression at time *t* was not predictive of SRPS at time *t+1* (Table 4). Similarly, MHS at time *t* was not predictive of SRPS at time *t+1*.

**Table 4:** Adjusted reverse-lagged associations between SRPS and subsequent mental health.

	Depression severity (HSCL as continuous scale)	Mental Health Status (MHS as continuous scale)
*Characteristic*	Regression estimate *b* (95% confidence interval [CI])	
*Sexual Relationship Power Scale (SRPS)*	−0.11 (−.027 to 0.04)	0.00 (−0.00 to 0.01)
***Socio-demographic Characteristics***		
Age	−0.01 (−0.05 to −0.07)	0.01 (−0.06 to 0.07)
Married	0.22 (0.06 to .038)**	0.23 (0.07 to 0.39)**
Household size	0.01 (−0.02 to 0.04)	0.01 (−0.02 to 0.04)
Educated more than secondary school	0.11 (−0.06 to 0.28)	0.12 (−0.06 to 0.29)
Unemployed	−0.14 (−0.31 to 0.02)	−0.14 (−0.30 to 0.02)
Distance to Clinic	0.07 (−0.02 to 0.17)	0.07 (−0.03 to 0.17)
***Clinical Characteristics***		
Heavy drinking (AUDIT-C)	0.18 (−0.14 to 0.50)	0.19 (−0.13 to 0.52)
Tobacco Use	0.20 (−0.05 to 0.44)	0.20 (−0.05 to 0.44)
WHO Stage IV clinical condition	−0.08 (−0.25 to 0.09)	−0.08 (−0.25 to 0.08)
CD4 count	−0.01 (−0.07 to 0.04)	−0.02 (−0.08 to 0.39)
Previously treated with ART	0.05 (−0.13 to 0.23)	−0.07 (−0.12 to .025)
*Constant*	*2.47*	*2.18*

* p<0.05,

** p<0.01,

*** p<0.001.

HSCL: Hopkins Symptomatic Check List; MHS: Mental Health Status; AUDIT-C: Alcohol Use Disorders Identification Test; WHO: World Health Organization; ART: Antiretroviral Treatment.

## Discussion

We found high levels of depression among rural Ugandan HIV-infected women, with more than one-quarter of participants meeting clinical criteria for probable depression, consistent with previous literature from the region [3], [4]. The prevalence of depression in our sample (23.7%) was lower than in another Ugandan study (47%) in which participants were ART-naïve [1], consistent with considerable evidence that HAART may have psychological benefits for patients [57]–[59]. Women with higher sexual relationship power had reduced depression symptom severity, were less likely to meet criteria for probable depression, and had better mental health status compared to women with low relationship power. These associations were statistically significant and large in magnitude.

Our confidence in the directionality of the interpretation was strengthened by the lagged and reverse-lagged analyses, in which we showed that SRPS at time *t* was predictive of depressive symptoms (HSCL) at time *t+1* but that depression at time *t* was not predictive of SRPS at time *t+1*. The secondary outcome of MHS exhibited similar patterns in lagged and reverse-lagged models. These data suggest that sexual relationship power may be predictive of subsequent mental health among HIV-infected women.

Our findings are consistent with the Theory of Learned Helplessness [60], which posits that self-esteem, cognition, and motivation are shaped by beliefs of personal control and perceived power over life's outcomes [61], [62]. Learned helplessness, in turn, has long been associated with depression in clinical populations [63]–[65], and recent studies in animal models have begun to establish its pathophysiology [66]–[70]. Likewise, the Theory of Gender and Power [44], which postulates that unequal power dynamics (in economic, decision-making, and emotional realms) limit the ability of to women to exercise personal control in relationships [71], provides a strong theoretical underpinning to the associations we observed in our data. This theory has been applied to a range of health outcomes, including HIV risk [42], [72]–[75], and intimate partner violence [29], but has yet to be explored among HIV-infected women, and has rarely been applied to mental health [76], [77]. This research suggests an important area for future research and intervention development among HIV-infected, female populations.

We attempted to determine whether different aspects of relationship power were differentially associated with depression but found that both SRPS subscales had statistically significant associations with the outcomes. Previous findings regarding SRPS subscales have been mixed, with many authors omitting Decision-Making Dominance due to low reliability, and others finding that only the Relationship Control sub-scale influenced health outcomes [42], [78], [79]. In-depth, qualitative research could further delineate the mechanisms through which sexual power may affect mental health.

Our study had several limitations. First, our measure of depression is based on a screening tool, and does not provide a conclusive diagnosis of major depressive disorder. Second, prospective longitudinal studies using longer follow-up times (beyond 3 months) and repeated measures are needed to confirm our findings. Third, our sample was limited to women who were initiating ART. Because individuals who are receiving ART have already overcome significant barriers to engagement in care, our findings may not be generalizeable to untreated populations.

Despite these limitations, our findings have implications for designing effective interventions for the mental health of HIV-positive women. The high burden of disease and dearth of evidence-based mental health interventions in low-resource settings [80] make intervention development a priority [81]–[83]. While HIV-infected women are at higher risk of depression than their male counterparts [27], [84], according to a recent systematic review on this topic, few specific interventions have been developed for this population [85]. Our findings suggest that relationship power may be an important potential driver of depressive symptom severity among women living with HIV and AIDS, one of the most vulnerable and marginalized subgroups in low-resource settings [39]. Effective interventions to improve women's relationship power may have the added benefit of contributing to secondary prevention of HIV transmission, since low relationship power and gender-unequal norms have been linked to higher-risk sex [86], [87], inconsistent condom use [88]–[90], and multiple partnerships [78]. Interventions to empower women in intimate dyadic relationships may have mental health benefits and should be assessed using randomized study designs.

In conclusion, we found that sexual relationship power in a sample of women living with HIV in rural Uganda is strongly associated with symptoms of depression and worse mental health status. The overlapping epidemics of HIV/AIDS and depression require integrated programs that target the intimate relationships shaping women's overall health and well being.

## References

[1] Kaharuza FM, Bunnell R, Moss S, Purcell DW, Bikaako-Kajura W, et al (2006) Depression and CD4 cell count among persons with HIV infection in Uganda. AIDS Behav 10: S105–111. 16802195 10.1007/s10461-006-9142-2

[2] Lundberg P, Rukundo G, Ashaba S, Thorson A, Allebeck P, et al (2011) Poor mental health and sexual risk behaviours in Uganda: A cross-sectional population-based study. BMC Public Health 11: 125. 21338500 10.1186/1471-2458-11-125 PMC3056745

[3] Ovuga E, Boardman J, Wasserman D (2005) The prevalence of depression in two districts of Uganda. Soc Psychiatry Psychiatr Epidemiol 40: 439–445. 16003593 10.1007/s00127-005-0915-0

[4] Kinyanda E, Woodburn P, Tugumisirize J, Kagugube J, Ndyanabangi S, et al (2011) Poverty, life events and the risk for depression in Uganda. Soc Psychiatry Psychiatr Epidemiol 46: 35–44. 19916062 10.1007/s00127-009-0164-8 PMC3432478

[5] Bolton P, Wilk CM, Ndogoni L (2004) Assessment of depression prevalence in rural Uganda using symptom and function criteria. Soc Psychiatry Psychiatr Epidemiol 39: 442–447. 15205728 10.1007/s00127-004-0763-3

[6] Gielen AC, McDonnell KA, Burke JG, O'Campo P (2000) Women's lives after an HIV-positive diagnosis: disclosure and violence. Matern Child Health J 4: 111–120. 10994579 10.1023/a:1009522321240

[7] Prachakul W, Grant JS, Keltner NL (2007) Relationships among functional social support, HIV-related stigma, social problem solving, and depressive symptoms in people living with HIV: a pilot study. J Assoc Nurses AIDS Care 18: 67–76. 10.1016/j.jana.2007.08.002 17991600

[8] Wu DY, Munoz M, Espiritu B, Zeladita J, Sanchez E, et al (2008) Burden of depression among impoverished HIV-positive women in Peru. J Acquir Immune Defic Syndr 48: 500–504. 18614919 10.1097/QAI.0b013e31817dc3e9

[9] Simbayi LC, Kalichman S, Strebel A, Cloete A, Henda N, et al (2007) Internalized stigma, discrimination, and depression among men and women living with HIV/AIDS in Cape Town, South Africa. Soc Sci Med 64: 1823–1831. 17337318 10.1016/j.socscimed.2007.01.006 PMC4271649

[10] Tsai AC, Bangsberg DR, Frongillo EA, Hunt PW, Muzoora C, et al (2012) Food insecurity, depression and the modifying role of social support among people living with HIV/AIDS in rural Uganda. Soc Sci Med 74: 2012–2019. 22513248 10.1016/j.socscimed.2012.02.033 PMC3348339

[11] Ciesla JA, Roberts JE (2001) Meta-analysis of the relationship between HIV infection and risk for depressive disorders. Am J Psychiatry 158: 725–730. 11329393 10.1176/appi.ajp.158.5.725

[12] Mello VA, Segurado AA, Malbergier A (2010) Depression in women living with HIV: clinical and psychosocial correlates. Arch Womens Ment Health 13: 193–199. 19760048 10.1007/s00737-009-0094-1

[13] Millikin CP, Rourke SB, Halman MH, Power C (2003) Fatigue in HIV/AIDS is associated with depression and subjective neurocognitive complaints but not neuropsychological functioning. J Clin Exp Neuropsychol 25: 201–215. 12754678 10.1076/jcen.25.2.201.13644

[14] Eller LS (2001) Quality of life in persons living with HIV. Clin Nurs Res 10: 401–423. 11881951 10.1177/C10N4R6

[15] Valente SM (2003) Depression and HIV disease. J Assoc Nurses AIDS Care 14: 41–51. 10.1177/1055329002250993 12698765

[16] Tsai AC, Weiser SD, Petersen ML, Ragland K, Kushel MB, et al (2010) A marginal structural model to estimate the causal effect of antidepressant medication treatment on viral suppression among homeless and marginally housed persons with HIV. Arch Gen Psychiatry 67: 1282–1290. 21135328 10.1001/archgenpsychiatry.2010.160 PMC3208399

[17] Cook JA, Cohen MH, Burke J, Grey D, Anastos K, et al (2002) Effects of depressive symptoms and mental health quality of life on use of highly active antiretroviral therapy among HIV-seropositive women. J Acquir Immune Defic Syndr 30: 401–409. 12138346 10.1097/00042560-200208010-00005

[18] Burack JH, Barrett DC, Stall RD, Chesney MA, Ekstrand ML, et al (1993) Depressive symptoms and CD4 lymphocyte decline among HIV-infected men. JAMA 270: 2568–2573. 7901433

[19] Evans DL, Ten Have TR, Douglas SD, Gettes DR, Morrison M, et al (2002) Association of depression with viral load, CD8 T lymphocytes, and natural killer cells in women with HIV infection. Am J Psychiatry 159: 1752–1759. 12359683 10.1176/appi.ajp.159.10.1752

[20] Ickovics JR, Hamburger ME, Vlahov D, Schoenbaum EE, Schuman P, et al (2001) Mortality, CD4 cell count decline, and depressive symptoms among HIV-seropositive women: longitudinal analysis from the HIV Epidemiology Research Study. JAMA 285: 1466–1474. 11255423 10.1001/jama.285.11.1466

[21] Antelman G, Kaaya S, Wei R, Mbwambo J, Msamanga GI, et al (2007) Depressive symptoms increase risk of HIV disease progression and mortality among women in Tanzania. J Acquir Immune Defic Syndr 44: 470–477. 17179766 10.1097/QAI.0b013e31802f1318 PMC6276368

[22] Cook JA, Grey D, Burke J, Cohen MH, Gurtman AC, et al (2004) Depressive symptoms and AIDS-related mortality among a multisite cohort of HIV-positive women. Am J Public Health 94: 1133–1140. 15226133 10.2105/ajph.94.7.1133 PMC1448411

[23] James JS (2004) Chronically depressed women with HIV almost twice as likely as others to die from AIDS-related causes; those with mental-health services had half the death rate of those without. AIDS Treat News: 2–3. 15386852

[24] Cohen MH, Fabri M, Cai X, Shi Q, Hoover DR, et al (2009) Prevalence and predictors of posttraumatic stress disorder and depression in HIV-infected and at-risk Rwandan women. J Womens Health (Larchmt) 18: 1783–1791. 19951212 10.1089/jwh.2009.1367 PMC2828188

[25] Stein MD, Hanna L (1997) Use of mental health services by HIV-infected women. J Womens Health 6: 569–574. 9356980 10.1089/jwh.1997.6.569

[26] Valverde EE, Purcell DW, Waldrop-Valverde D, Malow R, Knowlton AR, et al (2007) Correlates of depression among HIV-positive women and men who inject drugs. J Acquir Immune Defic Syndr 46 Suppl 2: S96–100. 18089990 10.1097/QAI.0b013e318157683b

[27] Nduna M, Jewkes RK, Dunkle KL, Shai NP, Colman I (2010) Associations between depressive symptoms, sexual behaviour and relationship characteristics: a prospective cohort study of young women and men in the Eastern Cape, South Africa. J Int AIDS Soc 13: 44. 21078150 10.1186/1758-2652-13-44 PMC2992477

[28] Smit J, Myer L, Middelkoop K, Seedat S, Wood R, et al (2006) Mental health and sexual risk behaviours in a South African township: a community-based cross-sectional study. Public Health 120: 534–542. 16684549 10.1016/j.puhe.2006.01.009

[29] Filson J, Ulloa E, Runfola C, Hokoda A (2010) Does powerlessness explain the relationship between intimate partner violence and depression? J Interpers Violence 25: 400–415. 19487687 10.1177/0886260509334401

[30] Ellsberg M, Jansen HA, Heise L, Watts CH, Garcia-Moreno C, et al (2008) Intimate partner violence and women's physical and mental health in the WHO multi-country study on women's health and domestic violence: an observational study. Lancet 371: 1165–1172. 18395577 10.1016/S0140-6736(08)60522-X

[31] Campbell R, Sullivan CM, Davidson I, William S (1995) Women who use domestic violence shelters: Changes in depression over time. Psychology of Women Quarterly 19: 237–255.

[32] Weiser SD, Tsai A, Senkungu J, Emenyonu N, Kawuma A, et al.. (2010) Effect of Low Sexual-Relationship Power on Viral Load Suppression among Women Receiving ART in Mbarara, Uganda. 17th Conference on Retroviruses and Opportunistic Infections. San Francisco, CA.

[33] Siedner MJ, Tsai AC, Dworkin S, Mukiibi NF, Emenyonu NI, et al (2012) Sexual Relationship Power and Malnutrition Among HIV-Positive Women in Rural Uganda. Behav 2012 Mar 2 [Epub ahead of print]. 10.1007/s10461-012-0162-9 PMC3439197 22382629

[34] Gove WR, Hughes M, Style CB (1983) Does marriage have positive effects on the psychological well-being of the individual? J Health Soc Behav 24: 122–131. 6886367

[35] Ross CE, Mirowsky J, Goldsteen K (1990) The impact of the family on health: The decade in review. Journal of Marriage and the Family: 1059–1078.

[36] Kawachi I, Berkman LF (2001) Social ties and mental health. J Urban Health 78: 458–467. 11564849 10.1093/jurban/78.3.458 PMC3455910

[37] Kim HK, McKenry PC (2002) The relationship between marriage and psychological well-being. Journal of Family Issues 23: 885–911.

[38] Kiecolt-Glaser JK, Fisher LD, Ogrocki P, Stout JC, Speicher CE, et al (1987) Marital quality, marital disruption, and immune function. Psychosom Med 49: 13–34. 3029796 10.1097/00006842-198701000-00002

[39] Farmer P, Connors M, Simmons J (1996) Women, poverty, and AIDS: sex, drugs, and structural violence: Common Courage Pr.

[40] Geng EH, Bwana MB, Kabakyenga J, Muyindike W, Emenyonu NI, et al (2010) Diminishing availability of publicly funded slots for antiretroviral initiation among HIV-infected ART-eligible patients in Uganda. PLoS One 5: e14098. 21124842 10.1371/journal.pone.0014098 PMC2991339

[41] Pulerwitz J, Gortmaker SL, DeJong W (2000) Measuring sexual relationship power in HIV/STD research. Sex Roles 42: 637–660.

[42] Dunkle KL, Jewkes RK, Brown HC, Gray GE, McIntryre JA, et al (2004) Gender-based violence, relationship power, and risk of HIV infection in women attending antenatal clinics in South Africa. Lancet 363: 1415–1421. 15121402 10.1016/S0140-6736(04)16098-4

[43] Ketchen B, Armistead L, Cook SL (2009) HIV infection, stressful life events, and intimate relationship power: The moderating role of community resources for black South African women. Women & health 49: 197. 19533510 10.1080/03630240902963648 PMC2704477

[44] Connell RW (1985) Theorising gender. Sociology 19: 260.

[45] Bolton P, Ndogoni L (2001) Cross-cultural assessment of trauma-related mental illness (Phase II): a report of research conducted by World Vision Uganda and The Johns Hopkins University.

[46] Martinez P, Andia I, Emenyonu N, Hahn JA, Hauff E, et al (2008) Alcohol use, depressive symptoms and the receipt of antiretroviral therapy in southwest Uganda. AIDS Behav 12: 605–612. 17968651 10.1007/s10461-007-9312-x PMC3591721

[47] Bolton P (2001) Cross-cultural validity and reliability testing of a standard psychiatric assessment instrument without a gold standard. J Nerv Ment Dis 189: 238–242. 11339319 10.1097/00005053-200104000-00005

[48] Epino HM, Rich ML, Kaigamba F, Hakizamungu M, Socci AR, et al (2012) Reliability and construct validity of three health-related self-report scales in HIV-positive adults in rural Rwanda. AIDS Care 10.1080/09540121.2012.661840 22428702

[49] Wu AW, Revicki DA, Jacobson D, Malitz FE (1997) Evidence for reliability, validity and usefulness of the Medical Outcomes Study HIV Health Survey (MOS-HIV). Qual Life Res 6: 481–493. 9330549 10.1023/a:1018451930750

[50] Wu AW, Rubin HR, Mathews WC, Ware JE Jr, Brysk LT, et al (1991) A health status questionnaire using 30 items from the Medical Outcomes Study. Preliminary validation in persons with early HIV infection. Med Care 29: 786–798. 1875745 10.1097/00005650-199108000-00011

[51] Shahriar J, Delate T, Hays RD, Coons SJ (2003) Commentary on using the SF-36 or MOS-HIV in studies of persons with HIV disease. Health Qual Life Outcomes 1: 25. 12914664 10.1186/1477-7525-1-25 PMC183842

[52] Revicki DA, Sorensen S, Wu AW (1998) Reliability and validity of physical and mental health summary scores from the Medical Outcomes Study HIV Health Survey. Med Care 36: 126–137. 9475468 10.1097/00005650-199802000-00003

[53] Babikako HM, Neuhauser D, Katamba A, Mupere E (2010) Feasibility, reliability and validity of health-related quality of life questionnaire among adult pulmonary tuberculosis patients in urban Uganda: cross-sectional study. Health Qual Life Outcomes 8: 93. 20813062 10.1186/1477-7525-8-93 PMC2944342

[54] Bradley KA, Bush KR, Epler AJ, Dobie DJ, Davis TM, et al (2003) Two brief alcohol-screening tests From the Alcohol Use Disorders Identification Test (AUDIT): validation in a female Veterans Affairs patient population. Arch Intern Med 163: 821–829. 12695273 10.1001/archinte.163.7.821

[55] Bush K, Kivlahan DR, McDonell MB, Fihn SD, Bradley KA (1998) The AUDIT alcohol consumption questions (AUDIT-C): an effective brief screening test for problem drinking. Ambulatory Care Quality Improvement Project (ACQUIP). Alcohol Use Disorders Identification Test. Arch Intern Med 158: 1789–1795. 9738608 10.1001/archinte.158.16.1789

[56] Derogatis LR, Lipman RS, Rickels K, Uhlenhuth EH, Covi L (1974) The Hopkins Symptom Checklist (HSCL). A measure of primary symptom dimensions. Mod Probl Pharmacopsychiatry 7: 79–110. 4607278 10.1159/000395070

[57] Low-Beer S, Chan K, Yip B, Wood E, Montaner JS, et al (2000) Depressive symptoms decline among persons on HIV protease inhibitors. J Acquir Immune Defic Syndr 23: 295–301. 10836751 10.1097/00126334-200004010-00003

[58] Rabkin JG, Ferrando SJ, Lin SH, Sewell M, McElhiney M (2000) Psychological effects of HAART: a 2-year study. Psychosom Med 62: 413–422. 10845355 10.1097/00006842-200005000-00015

[59] Palar K, Wagner G, Ghosh-Dastidar B, Mugyenyi P (2012) Role of antiretroviral therapy in improving food security among patients initiating HIV treatment and care in Uganda. AIDS 10.1097/QAD.0b013e328359b809 22948270

[60] Seligman ME (1974) Depression and learned helplessness.

[61] Abramson LY, Seligman ME, Teasdale JD (1978) Learned helplessness in humans: critique and reformulation. J Abnorm Psychol 87: 49–74. 649856

[62] Mirowsky J, Ross CE (1989) Social causes of psychological distress: Aldine de Gruyter.

[63] Grant RL (1978) The value of “learned helplessness” in understanding depression. Am J Psychiatry 135: 625. 10.1176/ajp.135.5.625a 645974

[64] Buchwald AM, Coyne JC, Cole CS (1978) A critical evaluation of the learned helplessness model of depression. J Abnorm Psychol 87: 180–193. 649851 10.1037//0021-843x.87.1.180

[65] Miller WR, Seligman ME (1975) Depression and learned helplessness in man. J Abnorm Psychol 84: 228–238. 1169264 10.1037/h0076720

[66] Li B, Piriz J, Mirrione M, Chung C, Proulx CD, et al (2011) Synaptic potentiation onto habenula neurons in the learned helplessness model of depression. Nature 470: 535–539. 21350486 10.1038/nature09742 PMC3285101

[67] Bougarel L, Guitton J, Zimmer L, Vaugeois JM, El Yacoubi M (2011) Behaviour of a genetic mouse model of depression in the learned helplessness paradigm. Psychopharmacology (Berl) 215: 595–605. 21340472 10.1007/s00213-011-2218-3

[68] Maier SF (2001) Exposure to the stressor environment prevents the temporal dissipation of behavioral depression/learned helplessness. Biol Psychiatry 49: 763–773. 11331084 10.1016/s0006-3223(00)01095-7

[69] Petty F, Kramer GL, Wu J, Davis LL (1997) Posttraumatic stress and depression. A neurochemical anatomy of the learned helplessness animal model. Ann N Y Acad Sci 821: 529–532. 9238245 10.1111/j.1749-6632.1997.tb48322.x

[70] Hajszan T, Dow A, Warner-Schmidt JL, Szigeti-Buck K, Sallam NL, et al (2009) Remodeling of hippocampal spine synapses in the rat learned helplessness model of depression. Biol Psychiatry 65: 392–400. 19006787 10.1016/j.biopsych.2008.09.031 PMC2663388

[71] Amaro H, Raj A (2000) On the margin: Power and women's HIV risk reduction strategies. Sex Roles 42: 723–749.

[72] Wingood GM, DiClemente RJ (2000) Application of the theory of gender and power to examine HIV-related exposures, risk factors, and effective interventions for women. Health Educ Behav 27: 539–565. 11009126 10.1177/109019810002700502

[73] Bermudez MP, Castro A, Gude F, Buela-Casal G (2010) Relationship power in the couple and sexual double standard as predictors of the risk of sexually transmitted infections and HIV: multicultural and gender differences. Curr HIV Res 8: 172–178. 20163348 10.2174/157016210790442669

[74] Hahm HC, Lee J, Rough K, Strathdee SA (2012) Gender power control, sexual experiences, safer sex practices, and potential HIV risk behaviors among young Asian-American women. AIDS Behav 16: 179–188. 21259042 10.1007/s10461-011-9885-2 PMC3389795

[75] Wingood GM, Scd, DiClemente RJ (2000) Application of the theory of gender and power to examine HIV-related exposures, risk factors, and effective interventions for women. Health Educ Behav 27: 539–565. 11009126 10.1177/109019810002700502

[76] Landstedt E, Asplund K, Gillander Gadin K (2009) Understanding adolescent mental health: the influence of social processes, doing gender and gendered power relations. Sociol Health Illn 31: 962–978. 19659740 10.1111/j.1467-9566.2009.01170.x

[77] Chonody JM, Siebert DC (2008) Gender Differences in Depression. Affilia 23: 338–348.

[78] Hahm HC, Lee J, Rough K, Strathdee SA (2011) Gender Power Control, Sexual Experiences, Safer Sex Practices, and Potential HIV Risk Behaviors Among Young Asian-American Women. AIDS Behav 10.1007/s10461-011-9885-2 PMC3389795 21259042

[79] Pettifor AE, Measham DM, Rees HV, Padian NS (2004) Sexual power and HIV risk, South Africa. Emerg Infect Dis 10: 1996–2004. 15550214 10.3201/eid1011.040252 PMC3328992

[80] Saraceno B, van Ommeren M, Batniji R, Cohen A, Gureje O, et al (2007) Barriers to improvement of mental health services in low-income and middle-income countries. Lancet 370: 1164–1174. 17804061 10.1016/S0140-6736(07)61263-X

[81] Chisholm D, Flisher AJ, Lund C, Patel V, Saxena S, et al (2007) Scale up services for mental disorders: a call for action. Lancet 370: 1241–1252. 17804059 10.1016/S0140-6736(07)61242-2

[82] Eaton J, McCay L, Semrau M, Chatterjee S, Baingana F, et al (2011) Scale up of services for mental health in low-income and middle-income countries. Lancet 378: 1592–1603. 22008429 10.1016/S0140-6736(11)60891-X

[83] Tomlinson M, Rudan I, Saxena S, Swartz L, Tsai AC, et al (2009) Setting priorities for global mental health research. Bull World Health Organ 87: 438–446. 19565122 10.2471/BLT.08.054353 PMC2686213

[84] Piccinelli M, Wilkinson G (2000) Gender differences in depression. Critical review. Br J Psychiatry 177: 486–492. 11102321 10.1192/bjp.177.6.486

[85] Sherr L, Clucas C, Harding R, Sibley E, Catalan J (2011) HIV and depression–a systematic review of interventions. Psychol Health Med 16: 493–527. 21809936 10.1080/13548506.2011.579990

[86] Yang X (2011) Prevalence and correlates of HIV unsafe sex and STIs among women working in China's entertainment industry. AIDS Care 23 Suppl 1: 75–82. 10.1080/09540121.2010.525620 PMC3156411 21660753

[87] Shannon K, Leiter K, Phaladze N, Hlanze Z, Tsai AC, et al (2012) Gender inequity norms are associated with increased male-perpetrated rape and sexual risks for HIV infection in Botswana and Swaziland. PLoS One 7: e28739. 22247761 10.1371/journal.pone.0028739 PMC3256140

[88] Pulerwitz J, Amaro H, De Jong W, Gortmaker SL, Rudd R (2002) Relationship power, condom use and HIV risk among women in the USA. AIDS Care 14: 789–800. 12511212 10.1080/0954012021000031868

[89] Weeks MR, Hilario H, Li J, Coman E, Abbott M, et al (2010) Multilevel social influences on female condom use and adoption among women in the urban United States. AIDS Patient Care STDS 24: 297–309. 20438372 10.1089/apc.2009.0312 PMC2933558

[90] Gagnon AJ, Merry L, Bocking J, Rosenberg E, Oxman-Martinez J (2010) South Asian migrant women and HIV/STIs: knowledge, attitudes and practices and the role of sexual power. Health Place 16: 10–15. 19747873 10.1016/j.healthplace.2009.06.009

